# By-products of apricot processing in quail feed: Effects on growth performance, carcass characteristics, and meat physicochemical quality

**DOI:** 10.14202/vetworld.2021.878-883

**Published:** 2021-04-12

**Authors:** Fatma Boubekeur, Rafik Arbouche, Yasmine Arbouche, Fodil Arbouche

**Affiliations:** 1 Department of Agronomy Science, Faculty of Life and Earth Sciences, University of Chadli Bendjedid El TARF 36000 Algeria; 2 Department of Agronomy, Faculty of Life and Earth Sciences, University of Ghardaia, Ghardaia 47000 Algeria; 3 Department of Agronomy, Faculty of Life Sciences, University of Sétif 1, El Bez, Sétif 19000, Algeria

**Keywords:** by-products, carcass, feed, growth performance, quails

## Abstract

**Background and Aim:**

The rearing of quails can have a stronger attraction for the breeders if we lower the cost prices by introducing by-products in their feed formulas. The aim of this study was to evaluate the effects of the partial substitution of soybean meal by apricot kernel cake (AKC) in the diet of quails, applied either sequentially or during all phases of rearing, on their growth performances, carcass characteristics, and meat physicochemical composition.

**Materials and Methods:**

A total of 600 one-day-old quails (*Coturnix coturnix japonica*), with equal sex ratio and weighing on average 7±0.2 g, were randomly distributed in one control group and three experimental groups, the latter being designed according to the rate of application of AKC in either sequential or non-sequential mode in different rearing phases. Each group was divided into five replicates of 30 quails, randomly distributed according to either substitution rate of soybean meal by the AKC (0%, 10%, 20%, or 30%) or farming phase.

**Results:**

The average daily gain from the 1^st^ to 45^th^ days (average daily gain_1–45d_) was found to be the highest (4.24 g/d/subject, p=0.021) for the 30% AKC-supplemented feed lot in either starter or finish incorporation (DF_TAA_), having an optimum final live weight of 193.4 g (p=0.028), a lowest feed conversion ratio of 3.08 (p=0.001), and a daily feed intake of 860 g (p=0.01). Carcass yield was recorded the highest (74.4%, p=0.02) with an optimum meat protein level (30.6%, p=0.024) and the lowest fat content (2.26%, p=0.001) for the same group as well.

**Conclusion:**

The partial substitution of soybean meal by AKC in the quails’ fattening feeding, during the finishing phase and for all rearing phases, led to a better growth performance, a better carcass yield, and an improved chemical composition of meat.

## Introduction

In Algeria, quail farming, or coturniculture, has attracted the attention of poultry farmers as a new avenue for livestock diversification and as a means of short-term production of meat and/or eggs, since quails are characterized by early sexual maturity and require low feed and space as compared with other poultry species [1,2]. Moreover, their rapid growth and a very little financial input are the reasons why the poorest rural households perceive quail farming as a complementary financial source for increasing people’s life quality [3,4]. However, as for most developing countries, Algeria is dependent on import of raw materials for the production of feed for domestic animals, resulting in too high costs for the poorest populations to provide the animal protein needs. According to Guermah *et al*. [5], the feed costs represent 60-70% of total expenditure in farms.

The introduction and use of local agro-industrial and agricultural by-products have been advocated by several authors [6-11] and the incorporation rates of these by-products depend on both a livestock species and a by-product type. The apricot kernel cake (AKC) is a by-product obtained after making apricot jams and juices whose 46,000-ha plantations are located in Hodna, a semi-arid area of Algeria, yielding annually around 290,000 tons [12]. According to El-Adawy *et al*. [13] and Ferradji *et al*. [14], the availability of almond cake is about 10,700 tons annually. According to Arbouche *et al*. [15], the crude protein content of AKC is about 42.3%, whereas its cellulose fiber amounts 7.7%. This meal has been introduced in feeding several species, including broiler chicken [16], fattening sheep [7], and rabbits [8,9,11] (alone or in mix with another by-product).

The aim of this study was to evaluate the effects of a partial substitution of soybean meal by apricot kernel meal in the quail diet, sequentially through all rearing phases, on their zootechnical performance, carcass characteristics, and the physicochemical composition of meat.

## Materials and Methods

### Ethical approval

The present study was conducted after approval of the Institutional Animal Ethics Committee of the Agriculture department of Ghardaia University, Algeria, with permission no. 0035/AEC/2019.

### Study location and period

To guarantee the maintenance of environmental conditions, the test was carried out in a 200-m^2^ building with a pad cooling system and fans in a professional quail breeding center (Wilaya of Sétif, Algeria), during the month of May 2019.

### Animals, food, and experimental protocol

A total of 600 one-day-old quails (*Coturnix japonica*), with an equal sex ratio and weighing an average of 7±0.2 g, were randomly assigned to four groups: One control and three experimental groups. The experimental groups were designed according to the rate of application of AKC in the different phases of rearing (Table-1). Each treatment contained 150 birds assigned into five replicates with 30 quails each, randomly distributed over 0.5 m^2^. They were kept on a litter made of crushed durum wheat straw, in a closed building with static ventilation. During the 1^st^ week, the average temperature was 39°C, and then it dropped to 36°C during the startup-growth phase and was further maintained at 24°C during the finishing phase. Continuous lighting was applied during the first 2 weeks. The chemical composition and nutritional value of AKC are shown in Table-2 [15]. The plans were formulated in accordance with the guidelines of the National Research Council (NRC 1994) (Table-3a and b).

**Table-1:** Arrangement of the different groups according to the rate of incorporation of apricot kernel cake (TAA) and the rearing phases.

Breeding phase	Starter-growth			Finishing		

	Incorporation rate					
**Control group (T)**	**0**			**0**		
**Experimental groups**
D_TAA_	**10**	**20**	**30**	0	0	0
F_TAA_	0	0	0	**10**	**20**	**30**
DF_TAA_	**10**	**20**	**30**	**10**	**20**	**30**

**Table-2:** Chemical composition of apricot kernel cake (% DM) [15].

Organic matter	96.70
Crude protein	42.30
Crude fiber	7.7
Ether extract	10.4
Crude ash	3.3
Nitrogen-free extract	36.7
NDF	18.4
ADF	10.7
ADL	7.4
Hemicellulose	7.7
Gross energy (kcal/kg DM)	5180
Metabolic energy* (kcal/kg DM)	4030
Lysine (g/100g feed)	1.8
Methionine (g/100g of food)	1.2
Cystine (g/100g of food)	1.3

DM=Dry matter

**Table-3a:** Formulas (kg/100 kg feed) of starter-growth (1-20 days) feeds to quail based on the rate of substitution of soybean meal by apricot kernel meal.

Composition	Control group	D_TAA_ group			F_TAA_ group			DF_TAA_ group		
			
	0	10	20	30	10	20	30	10	20	30
Corn	55	55	55	55	55	55	55	55	55	55
Soybean cake	36	32.4	28.8	25.2	36	36	36	32.4	28.8	25.2
Almond cake	0	**3.6**	**7.2**	**10.8**	0	0	0	**3.6**	**7.2**	**10.8**
Wheat bran	5	5	5	5	5	5	5	5	5	5
Limestone	1.8	1.8	1.8	1.8	1.8	1.8	1.8	1.8	1.8	1.8
Bi-calcium phosphorus	1.2	1.2	1.2	1.2	1.2	1.2	1.2	1.2	1.2	1.2
Mineral Vitamin supplement	1	1	1	1	1	1	1	1	1	1
**Calculated nutrient content**										
Metabolizable energy (kcal/kg DM)	2708	2870	2865	2870	2708	2708	2708	2870	2865	2870
Fat (c/o)	1.76	2.08	1.91	1.98	1.76	1.76	1.76	2.08	1.91	1.98
Crude protein (c/o)	20.8	21.7	23.7	26.6	20.8	20.8	20.8	21.7	23.7	26.6
Mineral materials	5.04	4.92	4.81	4.70	5.04	5.04	5.04	4.92	4.81	4.70
Raw cellulose	3.9	3.98	4.01	4.08	3.9	3.9	3.9	3.98	4.01	4.08
Lysine (c/o from PB)	3.44	3.53	3.47	3.49	3.44	3.44	3.44	3.53	3.47	3.49
Methionine (c/o from PB)	1.39	1.44	1.49	1.54	1.39	1.39	1.39	1.44	1.49	1.54

MVS=Mineral and vitamin supplement composed of: calcium: 150,700 mg/kg, sodium chloride: 332,000 mg/kg, Vitamin A: 800,000 UI, Vitamin D3: 150,000 UI, Vitamin E: 1500 mg/kg, Vitamin K: 200 mg/kg, Vitamin B1: 100 mg/ kg, Vitamin B2: 450 mg/kg, Vitamin B3: 780 mg/kg, Vitamin B6: 150 mg, Vitamin B12: 1 mg/kg, PP: 1000 mg/kg, folic acid: 50 mg/kg, biotin: 1.5 mg/kg, choline chloride: 35,000 mg/kg, iron: 3600 mg/kg, copper: 2250 mg/kg, zinc: 7500 mg/kg. The diets were formulated in accordance with the directives of the CNRC (1994). The figures in bold allow the reader to understand the sequential incorporation in the experimental farmings

**Table-3b:** Formulas (kg/100 kg feed) of finishing (21-45 days) feeds to quail based on the rate of substitution of soybean meal by apricot kernel meal.

Composition	Control group	D_TAA_ group			F_TAA_ group			DF_TAA_ group		
			
	0	10	20	30	10	20	30	10	20	30
Corn	62	62	62	62	62	62	62	62	62	62
Soybean cake	23	23	23	23	20.7	18.4	16.1	20.7	18.4	16.1
Almond cake	0	0	0	0	2.3	4.6	6.9	2.3	4.6	6.9
Wheat bran	12	12	12	12	12	12	12	12	12	12
Limestone	1.2	1.2	1.2	1.2	1.2	1.2	1.2	1.2	1.2	1.2
Bi-calcium phosphorus	0.8	0.8	0.8	0.8	0.8	0.8	0.8	0.8	0.8	0.8
Mineral Vitamin supplement	1	1	1	1	1	1	1	1	1	1
Metabolizable energy (kcal/kg DM)	2898	2898	2898	2898	2908	2904	2907	2908	2904	2907
Fat (c/o)	1.98	1.98	1.98	1.98	2.01	2.06	2.1	2.01	2.06	2.1
Crude protein (c/o)	16.7	16.7	16.7	16.7	18.7	19.7	21.7	18.7	19.7	21.7
Mineral materials	4.63	4.63	4.63	4.63	4.59	4.48	4.41	4.59	4.48	4.41
Raw cellulose	3.97	3.97	3.97	3.97	4.02	4.05	4.09	4.02	4.05	4.09
Lysine (c/o from PB)	3.23	3.23	3.23	3.23	3.01	2.96	2.92	3.01	2.96	2.92
Methionine (c/o from PB)	1.43	1.43	1.43	1.43	1.46	1.50	1.52	1.46	1.50	1.52

MVS=Mineral and vitamin supplement composed of: calcium: 150,700 mg/kg, sodium chloride: 332,000 mg/kg, Vitamin A: 800,000 UI, Vitamin D3: 150,000 UI, Vitamin E: 1500 mg/kg, Vitamin K: 200 mg/kg, Vitamin B1: 100 mg/kg, Vitamin B2: 450 mg/kg, Vitamin B3: 780 mg/kg, Vitamin B6: 150 mg, Vitamin B12: 1 mg/kg, PP: 1000 mg/kg, folic acid: 50 mg/kg, biotin: 1.5 mg/kg, choline chloride: 35,000 mg/kg, iron: 3600 mg/kg, copper: 2250 mg/kg, zinc: 7500 mg/kg. The diets were formulated in accordance with the directives of the CNRC (1994). The figures in bold allow the reader to understand the sequential incorporation in the experimental farmings

During all rearing phases, the feed was distributed *ad libitum* and the refusal was weighed daily. Water was dispensed at will. Only the control lot was vaccinated against both Newcastle disease and infectious bronchitis on the 7^th^ day of life. Individual animals were weighed on the day of reception and at the end of each rearing phase. Mortality rate, feed intake, live weight (LW), average daily gain (ADG), feed conversion ratio (FCR), and average daily intake (ADI) were determined.

### Carcass characteristics and physicochemical analysis of the meat

At the end of the finishing phase, ten quails were taken randomly from each replicate of each group and, after slaughtering, the carcasses, edible offal (heart, liver, and gizzard), legs, heads, and feathers were weighed. From each group, the meat was taken from carcasses, crushed, and homogenized, and the water content, mineral matter, protein, and fat were determined according to the methods of AOAC [17]. At 24 h postmortem, pH measurement was performed by direct insertion (~2 cm deep) of a pH meter electrode into the pectoral muscle of each quail according to the method reported earlier [18].

### Statistical analysis

Descriptive statistics and analysis of variance of the general linear one-way model were performed with Statistical Package for Social Science software (SPSS version 18) [IBM Corp., NY, USA] for the analysis of the following: LV; daily weight gain; feed intake; FCR; carcass, liver, gizzard, heart, head, leg, and feather weights; pH; water content; mineral matter; and protein and fat content of meat. A general linear model was used to test the effects of the factors on the variables, whereas the *post hoc* SNK (Student–Newman–Keules) test or Duncan test were used to estimate the significance or homogeneity between different subsets (test of comparison between means). The differences were considered significant with a 95% confidence interval.

## Results

### Growth parameters

Over the entire experimental period, the mortality rate remained zero regardless of the phase of AKC introduction in the quail diet. The incorporation of AKC had a positive effect (p<0.05) both on the LW recorded after 20 days and on the ADG_1–20d_ in all experimental groups (Table-4). The DF_TAA_ group with incorporation rates of 10%, 20%, and 30% increased LWs+6.8 g, +5.9 g, and +4 g, respectively, and showed ADG_1–20d_ values of 0.35, 0.31, and 0.21 g/d/subject, respectively. The same was observed for the D_TAA_ group. The F_TAA_ group had values identical to the control group for both LW and ADG_1–20d_ values.

**Table-4:** Effect of substitution of soybean meal with apricot kernel meal on quail weight growth (g) and average daily gain (ADG g/d/subject).

Groups	T	D_TAA_			F_TAA_			DF_TAA_			SEM	p
		
		10	20	30	10	20	30	10	20	30		
Starter-growth												
Initial weight	7	7	7	7	7	7	7	7	7	7		
Live weight at 20 d	73.8^b^	78.0^a^	79.6^a^	78.2^a^	72.6^b^	74.0^b^	73.2^b^	80.6^a^	79.7^a^	77.8^a^	2.516	0.001
ADG_1-20d_	3.52^b^	3.74^a^	3.82^a^	3.74^a^	3.45^b^	3.53^b^	3.48^b^	3.87^a^	3.83^a^	3.73^a^	0.500	0.001
Finishing phase												
Live weight at 45 d	177.8^c^	176.8^c^	179^c^	178.4^c^	186.4^b^	184.4^b^	185.8^b^	189.7^ab^	190.4^ab^	193.4^a^	0.360	0.028
ADG_21-45d_	4.16^b^	3.95^b^	3.98^b^	4.00^b^	4.55^ab^	4.42^ab^	4.50^ab^	4.54^ab^	4.61^ab^	4.81^a^	0.306	0.040
ADG_1-45d_	3.88^c^	3.86^c^	3.90^c^	3.89^c^	4.07^b^	4.03^b^	4.06^b^	4.15^ab^	4.17^ab^	4.24^a^	0.169	0.021

The indices indicate the period in days over which this parameter was calculated. The presence of different letters on the same line indicates a significant difference between diets (p<0.05)

In the finishing phase, at 45 days, the D_TAA_ group had both LW and ADG_21–45d_ values similar to those of the control group, regardless of the AKC incorporation rate introduced during the starter-growth phase. From these observations, it may be concluded that the incorporation of AKC only in the starter-growth phase had no influence on either LW or ADG values in the finishing phase. The incorporation of AKC in the final phase at 45 days significantly (p<0.05) improved both LW and ADG_21–45d_ values of the subjects in the F_TAA_ group averagely +7.7 g and +0.33 g/d/subject, respectively, as compared with those in the control group.

Considering the DF_TAA_ group, the 10% and 20% AKC-fed lots had both LWs at 45 days and ADG_21–45d_ values significantly (p<0.05) higher compared with either the control group or the D_TAA_ group, with increase of +12.25 g and +0.41 g/d/subject, respectively. In comparison with the control group, the 30% AKC-fed lot had significantly higher LW and ADG_21–45d_, with an improvement of +15.5 g and +0.65 g/d/subject, respectively. During all rearing phases, the ADG_1–45d_ values of both F_TAA_ and DF_TAA_ groups were significantly (p<0.05) higher than those of the control or DF_TAA_ group.

The daily feed intake of all experimental groups during the entire rearing period was significantly predominant in comparison with the control, regardless of the incorporation rate (p<0.001) (Table-5). The FCR of both the F_TAA_ and D_TAA_ groups had significantly less expressive values compared with that of both the D_TAA_ and control group and are in line with the obtained values for ADG and LW of these groups.

**Table-5:** Effects of substitution of soybean meal with apricot kernel cake on daily feed intake (DFI in g/subject) and feed conversion ratio of quail.

Groups	T	D_TAA_			F_TAA_			DF_TAA_			SEM	p-value
		
		10	20	30	10	20	30	10	20	30		
Daily feed intake												
1-20 d	230^c^	260^b^	277^b^	266^b^	242^c^	231^c^	235^c^	287^a^	278^a^	299^a^	1.65	0.001
21-45 d	405^c^	409^c^	403^c^	408^c^	512^b^	514^b^	518^b^	531^a^	536^a^	561^a^	1.52	0.001
1-45 d	635^d^	669^c^	680^c^	674^c^	754^b^	745^b^	753^b^	818^a^	814^a^	860^a^	1.02	0.01
Feed conversion ratio												
01-20 d	4.44^a^	3.49^b^	3.35^b^	3.55^b^	4.42^a^	4.54^a^	4.84^a^	3.12^c^	3.08^c^	3.04^c^	0.75	0.001
21-45 d	5.38^a^	5.42^a^	5.33^a^	5.20^a^	4.95^b^	4.78^b^	4.54^c^	3.33^c^	3.25^d^	3.10^d^	0.25	0.001
1-45 d	4.84^a^	4.80^a^	4.74^a^	4.55^a^	4.16^c^	4.14^c^	4.19^c^	3.2^d^	3.13^d^	3.08^d^	0.30	0.001

The presence of different letters on the same line indicates a significant difference between the diets (p<0.05)

### Carcass characteristics and meat physicochemical parameters

The incorporation of AKC meal as a substitute for soybean meal (10%, 20%, or 30%) for the D_TAA_ group during the starter-growth phase had no significant influence (p>0.05) on the carcass characteristics (Table-6). AKC introduced during the finishing phase and simultaneously in two rearing phases, exerted a significantly positive influence (p<0.05) on both carcass weight and carcass yield. At 30% substitution of soybean meal, AKC induced a maximum gain of +8.4% in carcass yield compared with the control group than when incorporated in all phases of rearing.

**Table-6:** Effects of substitution of soybean meal by apricot kernel meal on carcass characteristics and physicochemical parameters of quail meat.

Groups	T	D_TAA_			F_TAA_			DF_TAA_			SEM	p-value
		
		10	20	30	10	20	30	10	20	30		
Characteristics of the carcasses												
Live weight (g)	177^c^	176^c^	179^c^	178^c^	186^b^	184^b^	185^b^	189^ab^	190^ab^	194^a^	4.453	0.02
Carcass weight (g)	117.4^c^	116.3^c^	119.3^c^	118.0^c^	133.0^b^	134.3^b^	136^b^	137.6^ab^	139.0^ab^	144.3^a^	8.17	0.012
Carcass yield	66^c^	66^c^	66.6^c^	66^c^	71.5^b^	72,9^b^	73.5^b^	72.8^b^	73.1^b^	74.4^a^	1.02	0.02
Liver (g)	4.92	4.95	5.06	5.02	4.96	5.02	4.91	5.01	4.25	4.87	1.29	0.972
Liver/lw ratio	2.8	2.8	2.8	2.78	2.6	2.7	2.6	2.6	2.2	2.5	0.205	0.964
Gizzard (g)	2.78	2.56	2.78	2.57	2.87	2.64	2.78	2.62	2.34	2.65	0.240	0.859
Gizzard ratio/lw	1.44	1.33	1.48	1.32	1.48	1.36	1.49	1.43	1.24	1.40	0.172	0.856
Weight of the heart	1.73	1.54	1.41	1.29	1.40	1.56	1.28	1.53	1.35	1.56	0.133	0.377
Heart/lw ratio	0.89	0.80	0.75	0.67	0.72	0.81	0.68	0.82	0.72	0.83	0.10	0.281
Head weight (g)	6.25	7.68	7.50	7.24	7.80	7.35	7.07	7.73	7.30	7.54	0.987	0.197
Head of head ratio	3.23	3.98	3.98	3.75	4.02	3.78	3.78	4.13	3.86	3.98	0.652	0.128
Leg weight (g)	3.48	3.51	3.76	3.67	3.84	3.69	3.46	3.66	3.76	3.79	0.254	0.562
Paws/lw ratio	1.80	1.82	2.0	1.90	1.98	1.90	1.85	1.96	1.99	2.00	0.315	0.351
Feather weight (g)	27.8	23.8	27.1	25.9	24.1	27.1	27.9	25.6	26.2	24.8	0.814	0.562
Ratio of feathers/lw	14.4	12.33	14.41	13.42	12.42	13.97	14.92	13.69	13.86	13.12	0.672	0.728
Physico-chemical parameters of the meat												
pH 24 h post mortem	5.92^b^	5.96^b^	6.11^a^	6.06^a^	5.92^b^	6.04^a^	6.03^a^	5.98^b^	6.06^a^	6.00^a^	0.015	0.01
Moisture content	75.1^a^	75.1^a^	73.4^b^	72.6^b^	75.3^a^	74.0^b^	72.9^b^	71.0^c^	71.2^c^	71.0^c^	0.026	0.001
Mineral materials	1.32	1.36	1.18	1.28	1.58	1.73	1.41	1.00	1.05	1.08	0.183	0.125
Protein	25.0^b^	28.1^a^	29.6^a^	27.8^a^	27.6^a^	28.4^a^	30.1^a^	29.9^a^	30.2^a^	30.6^a^	1.12	0.024
Fats	5.61^a^	4.07^a^	4.45^b^	4.89^b^	3.32^b^	3.87^b^	3.53^b^	2.24^c^	2.25^c^	2.26^c^	0.015	0.001

The presence of different letters on the same line indicates a significant difference between the diets (p<0.05)

The mineral content of meat was found not to be influenced by the substitution rate, regardless of the rearing phase. The 24 h postmortem pH of the 10% AKC-fed lot in all experimental groups was identical to that of the control lot (p<0.05), but compared with the 20% and 30% lots, it showed significantly (p<0.02) higher (+0.13) pH values. Only the 20% and 30% lots in both the D_TAA_ and F_TAA_ groups had significantly lower water content compared with the control, whereas all lots in the DF_TAA_ group had lower pH values (p<0.001). For all experimental groups and their surrogate batches (lots), the meat protein content was significantly higher (+4.14 points) (p<0.014). The 10% lot in the F_TAA_ group contained the same amount of fat as that in the control group, whereas the 20% and 30% lots and all lots in the F_TAA_ group with similar values were significantly (p<0.001) less expressive (−2.0 points on average). All lots from the DF_TAA_ group showed either identical or lower values, with a decrease of −3.36 points in comparison with the control lot.

## Discussion

The incorporation of AKC as a substitute for soybean meal in the quail feed during the finishing phase (F_TAA_) and two rearing phases (DF_TAA_) resulted in an increase in LW and the ADI, while a decrease in the FCR was also recorded. This can be explained by an increase in the protein content of the experimental formulas as reported by Kouatcho *et al*. [19], who recommended this content to be of 27%, whereas Menassé [20] and Vali [21] reported higher protein requirements for quails than for broiler chicken. The LWs we recorded on the 45^th^ day remained below those reported by Kouatcho *et al*. [19] (219 g vs. 193 g) but higher than those recorded by Ayoola *et al*. [22] (140 g vs. 193 g) and similar to those noted by Tufan and Bolacali [23] (190 g).

Contrary to the observations published by several authors [20,21,24], who suggested that the increase in the protein levels in feed did not lead to an increase in the quantity ingested, our results led us to hypothesize that there might exist strains with different adult weights, which could be distinguished in heavy and light groups, and whose consumption would vary as pointed out by Alkan *et al*. [25] and Berrama *et al*. [26]. In addition, the improvement in the recorded FCR, particularly visible in the DF_TAA_ group, reflects better food assimilation, which is linked to a convenient feed protein level (21.7%) in this group [21]. However, the optimum protein level for the replacement, in participation above 30%, could lead to poor feed conversion, which further results in poor assimilation of nutrients as reported by Kita *et al*. [27] and Devlin [28]. The FCR values recorded in our study are similar to those scored by Bonos *et al*. [24] but are significantly lower than those reported by Kouatcho *et al*. [19].

Carcass yields are relatively more consistent within the F_TAA_ and DF_TAA_ groups, which is likely to be induced by the optimal protein levels in feed rations [29]; however, the obtained values are comparable with those reported by Sahin *et al*. [30] and Tufan and Bolacali [23] but higher than those scored by Alkan *et al*. [25].

A lower pH of muscles (<6) observed for the control and for the 10% lots in the experimental groups, as compared with the other lots, represents the main cause of the variation in meat quality [31]. In addition, Larzul and Gondret [29] found that low pH influences meat storability. According to Warris *et al*. [32], high values of the ultimate pH of meat, particularly poultry meat, are caused by low concentrations of glycogen measured immediately after slaughtering. In general, pH values are influenced by many factors such as age, sex, husbandry systems, feed additives, pre-slaughtering stress (which reflects on the hormonal status), muscle morphology, and glycogen content [33].

According to Benatmane [34], the ultimate pH, brightness, and color of carcasses are not influenced by diet. The increase in meat protein levels in the experimental groups’ batches is not explained by better protein assimilation from the feed rations [21]. The incorporation of AKC induces a decrease in fat content of the quail meat in proportions relative to its incorporation at the different rearing stages of the experimental groups. This observation is opposed to the results performed on rabbit meat by Ouzzir *et al*. [11] but is supported by the results of Mennani *et al*. [8,9], who applied a twin mixture of date scraps and AKC.

## Conclusion

The incorporation of AKC in feed formulas in coturniculture, whether or not sequentially applied, leads us to conclude that its incorporation, either in the finishing stage only or during the entire breeding, significantly influenced growth performance, carcass yield, and the meat chemical composition.

## Authors’ Contributions

FB: Prepared the ground conditions and collected the data. RA revised the manuscript. YA performed the analysis of the data. FA designed the study and drafted the manuscript. All authors have read and approved the final manuscript.
